# Supplementary material for: ProteinShader: illustrative rendering of macromolecules
Source: BMC Struct Biol. 2009 Mar 30;9:19. doi: 10.1186/1472-6807-9-19 (PMC2672931; doi:10.1186/1472-6807-9-19)
Supplement: Additional file 1 — ProteinShader program without source code. This compressed file contains the complete ProteinShader program including associated libraries, but no source code. A README.txt file gives an overview of the ProteinShader distribution, and the index.html file in the help subdirectory has directions on getting started with the program as well as a set of tutorials. [file 1472-6807-9-19-S1.zip › ProteinShader-beta-0_9_4-binary/help/api/org/proteinshader/gui/enums/class-use/DisplayEnum.html]

Uses of Class org.proteinshader.gui.enums.DisplayEnum (ProteinShader API)


|  |  |  |
| --- | --- | --- |
| **Overview** \| **Package** \| **Class** \| **Use** \| **Tree** \| **Deprecated** \| **Index** \| **Help** | |  |
| PREV   NEXT | **FRAMES**    **NO FRAMES**     **All Classes** |  |


---


## **Uses of Class org.proteinshader.gui.enums.DisplayEnum**

| Packages that use DisplayEnum | |
| --- | --- |
| **org.proteinshader.graphics.adapter** | Holds the StructureToGraphics class, which is used to manage the use of the drawing classes and OpenGL display lists. |
| **org.proteinshader.graphics.displaylists** | Holds the classes needed to manage OpenGL display lists, which are used to cache reusable geometry for spheres, cylinders, ribbon segments, and tube segments. |
| **org.proteinshader.gui** | Holds all of the Swing GUI components and their associated listeners, including class Renderer, which is registered as a listener for the GLCanvas object that is used a drawing surface. |
| **org.proteinshader.gui.components.controlpanel** | Holds the JPanels used in the right-side retractable control panel of the GUI. |
| **org.proteinshader.gui.components.menubar** | Holds the JMenus used in menu bar above the canvas. |
| **org.proteinshader.gui.enums** | Holds the enums needed by org.proteinshader.gui and its subpackages. |

Uses of DisplayEnum in org.proteinshader.graphics.adapter

| Methods in org.proteinshader.graphics.adapter that return DisplayEnum | |
| --- | --- |
| `DisplayEnum` | `StructureToGraphics.getDisplayType()`             Returns the current display type (TUBES, RIBBONS, RIBBONS\_AND\_TUBES, FRENET\_FRAMES, SPACE\_FILLING, BALLS\_AND\_STICKS, or STICKS). |

| Methods in org.proteinshader.graphics.adapter with parameters of type DisplayEnum | |
| --- | --- |
| `void` | `StructureToGraphics.cacheSegmentGeometry(GL gl, Model model, DisplayEnum displayType)`             Calls on the SegmentReferences object to cache OpenGL display lists for the Segments of the current Model. |
| `CylinderListInfo` | `StructureToGraphics.getCylinderInfo(DisplayEnum displayType)`             Returns the CylinderListInfo object that holds the information on an OpenGL display list for a cylinder that is currently used for drawing Bonds. |
| `SphereListInfo` | `StructureToGraphics.getSphereInfo(DisplayEnum displayType)`             Returns the SphereListInfo object that holds the information on an OpenGL display list for a sphere that is currently used for drawing Atoms. |
| `void` | `StructureToGraphics.setDisplayType(DisplayEnum displayType)`             Sets the display type (TUBES, RIBBONS, RIBBONS\_AND\_TUBES, FRENET\_FRAMES, SPACE\_FILLING, BALLS\_AND\_STICKS, or STICKS). |

Uses of DisplayEnum in org.proteinshader.graphics.displaylists

| Methods in org.proteinshader.graphics.displaylists that return DisplayEnum | |
| --- | --- |
| `DisplayEnum` | `GeometricListInfo.getDisplayType()`             Returns the display type that the geometric object is intended for: SPACE\_FILLING, BALLS\_AND\_STICKS, or STICKS. |

| Methods in org.proteinshader.graphics.displaylists with parameters of type DisplayEnum | |
| --- | --- |
| `void` | `SegmentReferences.cacheSegmentGeometry(GL gl, Model model, DisplayEnum displayType)`             Caches an OpenGL display list for each Segment of each Region object in the Model. |
| `void` | `GeometricListInfo.setDisplayType(DisplayEnum displayType)`             Sets the display type that this geometric object is intended for: SPACE\_FILLING, BALLS\_AND\_STICKS, or STICKS. |

Constructors in org.proteinshader.graphics.displaylists with parameters of type DisplayEnum

`CylinderListInfo(int displayListName,
DisplayEnum displayType,
double baseRadius,
double topRadius,
double height,
int slices,
int stacks,
boolean baseCapped,
boolean topCapped,
int capSlices,
int capStacks)`
  
          Constructs a CylinderListInfo.

`GeometricListInfo(int displayListName,
DisplayEnum displayType)`
  
          Constructs a GeometricListInfo.

`SegmentListInfo(int displayListName,
DisplayEnum displayType)`
  
          Constructs a SegmentListInfo.

`SphereListInfo(int displayListName,
DisplayEnum displayType,
double radius,
int slices,
int stacks)`
  
          Constructs a SphereListInfo.

Uses of DisplayEnum in org.proteinshader.gui

| Methods in org.proteinshader.gui that return DisplayEnum | |
| --- | --- |
| `DisplayEnum` | `MediatorImpl.getDisplayType()`             Returns the current display type (SPACE\_FILLING, BALLS\_AND\_STICKS, or STICKS). |
| `DisplayEnum` | `Mediator.getDisplayType()`             Returns the current display type (SPACE\_FILLING, BALLS\_AND\_STICKS, or STICKS). |

| Methods in org.proteinshader.gui with parameters of type DisplayEnum | |
| --- | --- |
| `CylinderListInfo` | `MediatorImpl.getCylinderInfo(DisplayEnum displayType)`             Returns the CylinderListInfo object that holds the information on an OpenGL display list for a cylinder that is currently used for drawing Bonds. |
| `CylinderListInfo` | `Mediator.getCylinderInfo(DisplayEnum displayType)`             Returns the CylinderListInfo object that holds the information on an OpenGL display list for a cylinder that is currently used for drawing Bonds. |
| `SphereListInfo` | `MediatorImpl.getSphereInfo(DisplayEnum displayType)`             Returns the SphereListInfo object that holds the information on an OpenGL display list for a sphere that is currently used for drawing Atoms. |
| `SphereListInfo` | `Mediator.getSphereInfo(DisplayEnum displayType)`             Returns the SphereListInfo object that holds the information on an OpenGL display list for a sphere that is currently used for drawing Atoms. |
| `void` | `MediatorImpl.setDisplayType(DisplayEnum displayType)`             Sets the current display type (SPACE\_FILLING, BALLS\_AND\_STICKS, or STICKS). |
| `void` | `Mediator.setDisplayType(DisplayEnum displayType)`             Sets the current display type (SPACE\_FILLING, BALLS\_AND\_STICKS, *etc*. |
| `void` | `Renderer.setVisibleDrawables(Model model, DisplayEnum displayType)`             An array of opaque Segments, and array of opaque Atoms and Bonds, and an array of translucent Drawable objects will be obtained from the Model. |

Uses of DisplayEnum in org.proteinshader.gui.components.controlpanel

| Methods in org.proteinshader.gui.components.controlpanel with parameters of type DisplayEnum | |
| --- | --- |
| `void` | `ControlPanel.updateAtomScalePanel(DisplayEnum displayType)`             Tells the ModifierPanel to update the AtomScalePanel with the current display type. |
| `void` | `ModifierPanel.updateAtomScalePanel(DisplayEnum displayType)`             Updates the AtomScalePanel so that it knows the current display type. |
| `void` | `AtomScalePanel.updateDisplayType(DisplayEnum displayType)`             Changes will be applied only if the display type is SPACE\_FILLING or BALLS\_AND\_STICKS. |

Uses of DisplayEnum in org.proteinshader.gui.components.menubar

| Fields in org.proteinshader.gui.components.menubar declared as DisplayEnum | |
| --- | --- |
| `static DisplayEnum[]` | `DisplayMenu.cartoonSubmenu`             This list determines which menu items are placed in the 'Cartoon' submenu (currently Tubes, Ribbons, and Frenet Frames). |
| `static DisplayEnum` | `DisplayMenu.INITIAL_DISPLAY_TYPE`             The display type to set as selected in the 'Display' menu. |

| Methods in org.proteinshader.gui.components.menubar that return DisplayEnum | |
| --- | --- |
| `DisplayEnum` | `DisplayMenu.setToDefault()`             Sets the "Display" menu to the default INITIAL\_DISPLAY\_TYPE. |

Uses of DisplayEnum in org.proteinshader.gui.enums

| Methods in org.proteinshader.gui.enums that return DisplayEnum | |
| --- | --- |
| `static DisplayEnum` | `DisplayEnum.getDefaultDisplayType()`             Returns the default display type, which is BALLS\_AND\_STICKS. |
| `static DisplayEnum` | `DisplayEnum.valueOf(String name)`             Returns the enum constant of this type with the specified name. |
| `static DisplayEnum` | `DisplayEnum.valueOfMenuName(String menuName)`             Returns the DisplayEnum with the same menu name as the String given as an argument. |
| `static DisplayEnum[]` | `DisplayEnum.values()`             Returns an array containing the constants of this enum type, in the order they're declared. |

---


|  |  |  |
| --- | --- | --- |
| **Overview** \| **Package** \| **Class** \| **Use** \| **Tree** \| **Deprecated** \| **Index** \| **Help** | |  |
| PREV   NEXT | **FRAMES**    **NO FRAMES**     **All Classes** |  |


---

# *Copyright © 2007-2008*
